# Results of a previously unreported extravesical ureteroneocystostomy technique without ureteral stenting in 500 consecutive kidney transplant recipients

**DOI:** 10.1371/journal.pone.0244248

**Published:** 2021-01-11

**Authors:** Gaetano Ciancio, Ahmed Farag, Javier Gonzalez, Paolo Vincenzi, Jeffrey J. Gaynor

**Affiliations:** 1 Department of Surgery, University of Miami Miller School of Medicine, Jackson Memorial Hospital, Miami, Florida, United States of America; 2 Department of Urology, University of Miami Miller School of Medicine, Jackson Memorial Hospital, Miami, Florida, United States of America; 3 Miami Transplant Institute, University of Miami Miller School of Medicine, Jackson Memorial Hospital, Miami, Florida, United States of America; 4 Department of Surgery, Zagazig University School of Medicine, Zagazig, Egypt; 5 Department of Urology, Hospital General Universitario Gregorio Marañón, Madrid, Spain; Medical University of Gdansk, POLAND

## Abstract

Urologic complications can still occur following kidney transplantation, sometimes requiring multiple radiological and/or surgical procedures to fully correct the problem. Previously proposed extravesical ureteral reimplantation techniques still carry non-negligible risks of the patient developing urologic complications. About 10 years ago, a new set of modifications to the Lich-Gregoir technique was developed at our center, with the goal of further minimizing the occurrence of urologic complications, and without the need for initial ureteral stent placement. It was believed that an improvement in the surgical technique to minimize the risk of developing urologic complications was possible without the need for stent placement at the time of transplant. In this report, we describe the advantages of this technique (i.e., mobilized bladder, longer spatulation of the ureter, inclusion of bladder mucosa with detrusor muscle layer in the ureteral anastomosis, and use of a right angle clamp in the ureteral orifice to ensure that it does not become stenosed). We also retrospectively report our experience in using this technique among 500 consecutive (prospectively followed) kidney transplant recipients transplanted at our center since 2014. During the first 12mo post-transplant, only 1.4%(7/500) of patients developed a urologic complication; additionally, only 1.0%(5/500) required surgical repair of their original ureteroneocystostomy. Five patients(1.0%) developed a urinary leak, with 3/5 having distal ureteral necrosis, and 1/5 subsequently developing a ureteral stricture. Two other patients developed ureteral stenosis, one due to stricture and one due to ureteral stones. These overall results are excellent when compared with other reports in the literature, especially those in which routine stenting was performed. In summary, we believe that the advantages in using this modified extravesical ureteroneocystostomy technique clearly help in lowering the early post-transplant risk of developing urologic complications. Importantly, these results were achieved without the need for ureteral stent placement at the time of transplant.

## Introduction

Despite advances in surgical techniques in the field of kidney transplantation, urologic complications, i.e., ureterovesical junction (UVJ) stenosis or stricture, ureteral necrosis, and urinary leak, may still occur in the early post-transplant period and are associated with a myriad of potentially unfavorable outcomes, including recurring morbidity, repeat hospitalizations, graft failure, and even death [1, 2] Some urologic complications may require multiple radiological and/or surgical procedures to fully correct the problem. The reported incidence of urologic complications following renal transplantation in defined cohorts of at least 200 patients ranges between 1.7%-15.0% [1]. Potential risk factors for their development include older donor age, a pre-existing comorbidity in the recipient (e.g., diabetes mellitus, cardiovascular disease), and iatrogenic ureteral injury [2]. In fact, the transplanted ureter continues to be the major culprit behind the development of most of these urologic complications.

Multiple techniques for performing the ureteral anastomosis have been introduced over the years with a major goal of further reducing the patient’s risk of developing a urologic complication post-transplant. The two most commonly used techniques include the intravesical technique described by Politano-Leadbetter [3] and the extravesical technique described by Lich-Gregoir [4, 5]. The older Politano-Leadbetter technique requires a second cystotomy and longer donor ureter, and its use is known to be associated with some hematuria [6]. Advantages in performing the extravesical ureteral anastomosis are well known and include avoidance of a separate cystostomy (thereby decreasing operative time), a lowered risk of developing hematuria, an enhanced performance capability with a shorter donor ureter, and a reduced time required for Foley catheter drainage [6].

Modifications to the original extravesical approach of Lich-Gregoir were proposed as improvements to this technique [7–15]. However, each of these extravesical ureteral reimplantation techniques still carry non-negligible risks of the patient developing urologic complications [6, 16]. It has also been argued by some that in order to minimize urologic complication risk, a ureteral stent should be routinely placed at the time of transplant [1, 2, 6, 17].

About 10 years ago, a new set of modifications to the extravesical Lich-Gregoir technique was developed at the Miami Transplant Institute with the goal of further minimizing and potentially even completely avoiding the occurrence of any urologic complications, and without the need for initial ureteral stent placement. It was believed that if the surgical technique could be sufficiently improved so as to avoid/minimize urologic complication risk post-transplant, then there would be no need for routine or even selective stent placement at the time of transplant. In this report, we describe this technique and retrospectively report our experience using it among 500 consecutive (prospectively followed) kidney transplant recipients transplanted at our center since 2014. We additionally provide a descriptive comparison of our results with those reported in the literature using the extravesical Lich-Gregoir or modified Lich-Gregoir technique with and without routine stent placement.

## Materials and methods

### Surgical technique

In comparison with all of the previously published modifications [7–15] to the Lich-Gregoir extravesical ureteroneocystostomy [4, 5], our approach includes certain technical aspects of the others along with certain aspects that are different. Once the kidney allograft is anastomosed to the external iliac vessels, the bladder is mobilized to facilitate upward mobility (with the kidney) following closure of the abdominal wall. To facilitate dissection and mobilization, the bladder is gravity filled with normal saline containing antibiotic solution with Foley catheter clamping. The umbilico-vesical fascia is then opened, allowing better expansion and mobilization of the bladder [18]. After renal allograft reperfusion, attention is paid if there was a ureteral blood flow demarcation and to avoid ureteral rotation or kinking. Then, the transplanted ureter is trimmed, and a tension-free anastomosis is performed with mobilization of the bladder, allowing the ureter and bladder to move upward with the kidney in case this occurs during abdominal wall closure.

**Fig 1:**
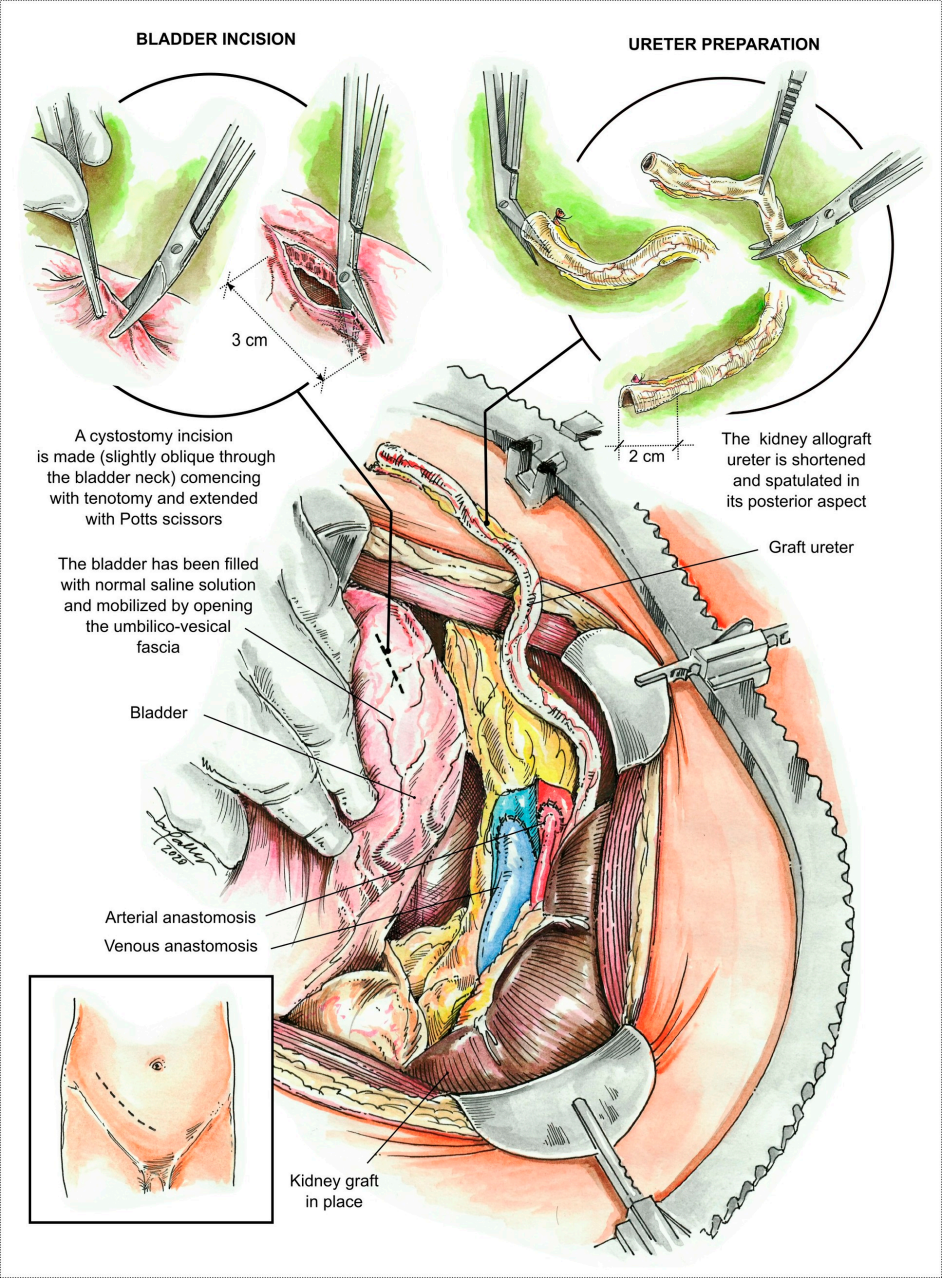
Visual of the arterial anastomosis, venous anastomosis, bladder incision, and ureteral preparation. Bladder incision is 3cm, and the ureteral spatulation is 2cm.

The extravesical ureteral anastomosis starts with a 3cm cystostomy on the anterolateral wall of the bladder dome, exposing the bladder mucosa with some detrusor layer (BMDL). The direction of incision is slightly oblique going towards the bladder neck (Fig 1). Opening of the BMDL is made with tenotomy and extended with a Potts scissors. The graft ureter is then shortened and posteriorly spatulated, being about 2cm long (Fig 1).

**Fig 2:**
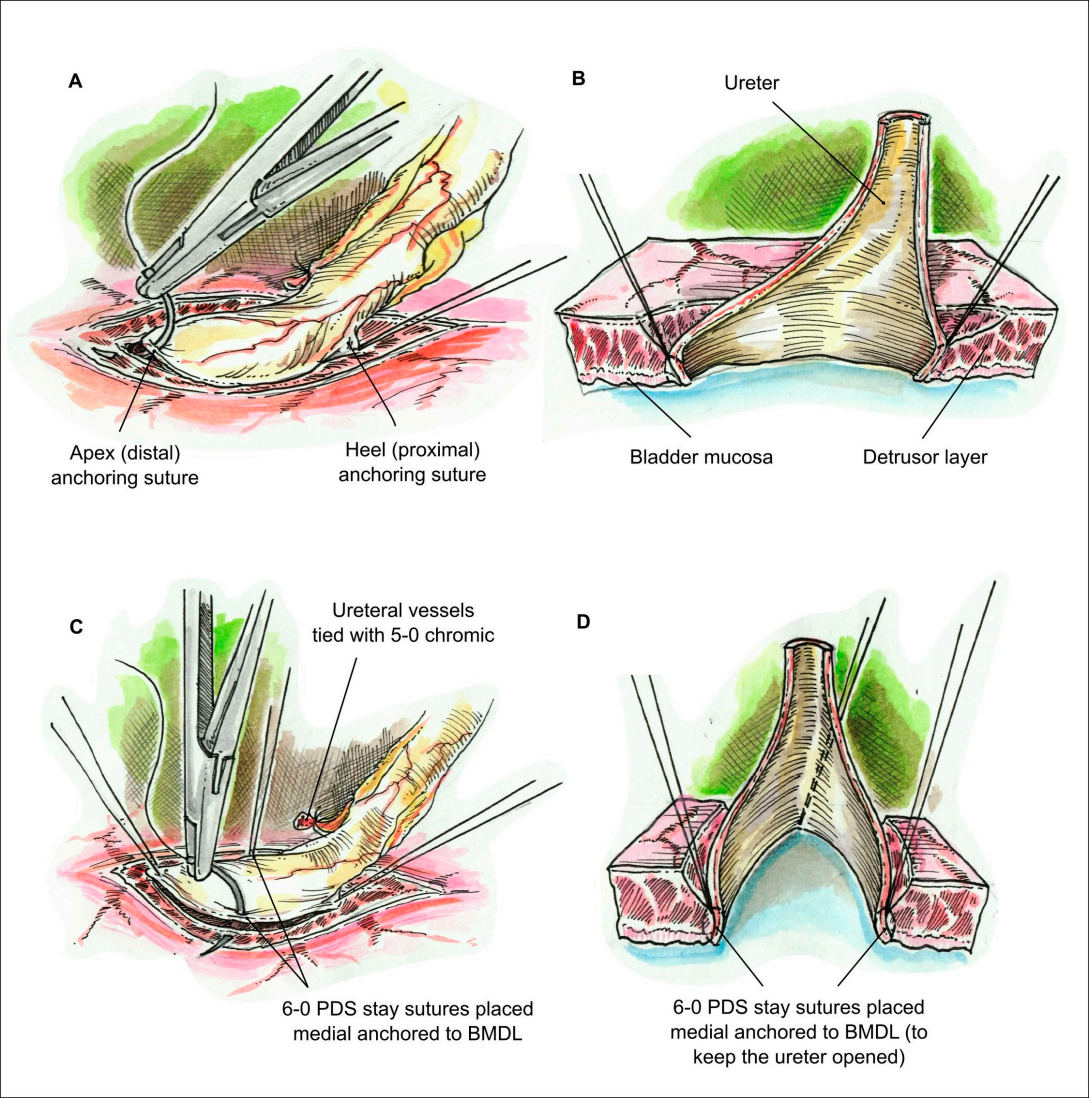
Visual of the heel and apex suturing of the ureter so as to anchor it medially to the bladder mucosa with some detrusor layers (BMDL) while also keeping it open. A) Placing the suture at the apex and heel of the ureter; B) Cross-section showing the suture including BMDL; C) Placing both anchor sutures at either site including BMDL. D) Cross-section showing the wide opening of the transplanted ureter.

Ureteral vessels are tied with 5–0 chromic. The ureter to BMDL anastomosis is performed using a 6–0 polydioxanone suture (PDS) starting from the heel of the ureter. The heel (proximal) and apex (distal) of the ureter are anchored to BMDL at almost full thickness. Two stay sutures with 6–0 PDS are placed medial and lateral, then anchored to BMDL in order to keep the ureter open (Fig 2).

**Fig 3:**
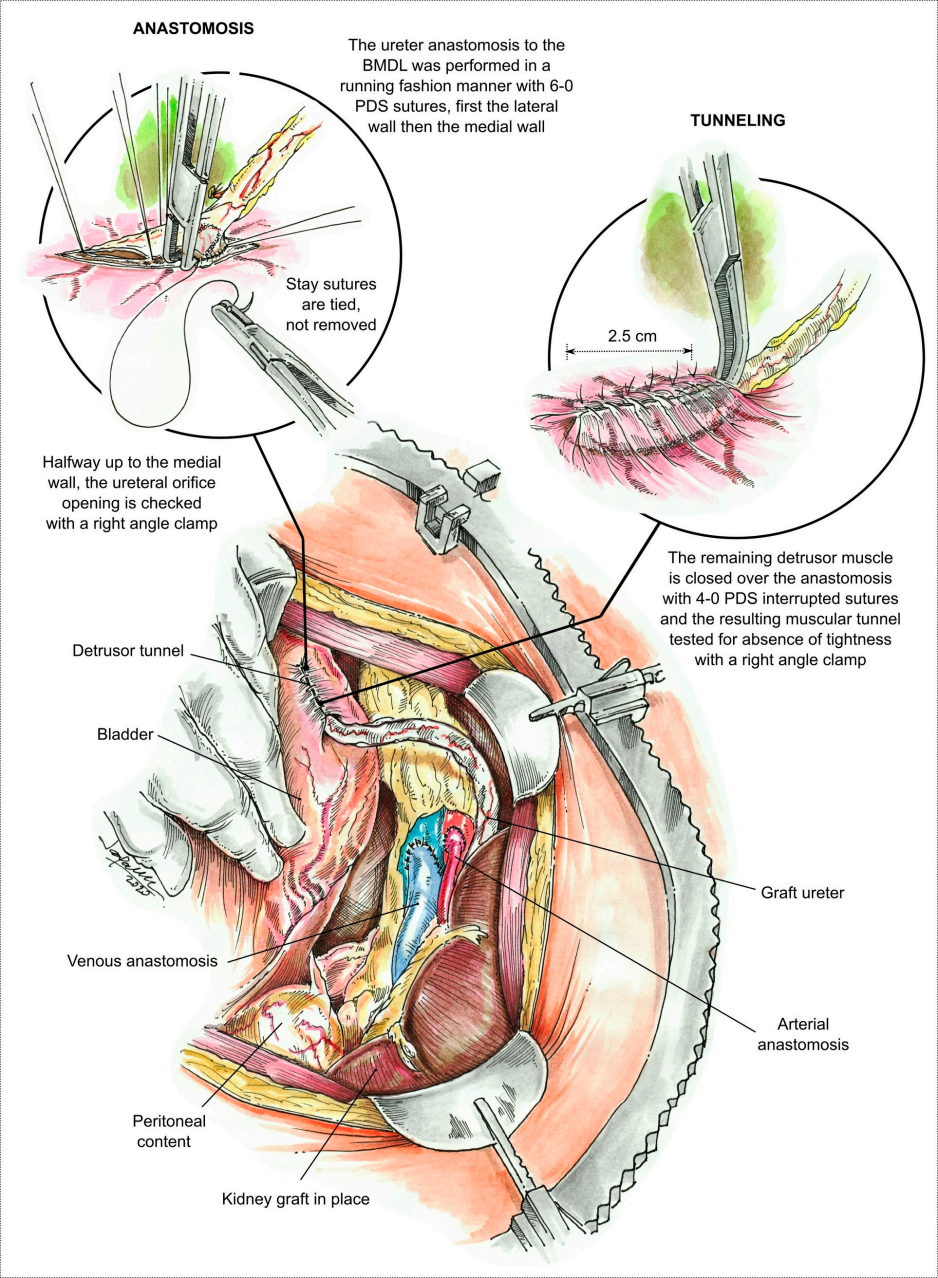
Visual of the ureteral anastomosis to the bladder mucosa with some detrusor layers BMDL and creation of an anti-reflux tunnel using the remaining detrusor muscle. The upper left circle (Anastomosis) shows the right angle introduced into the ureteral opening to ensure that it is not stenosed or incorporated by the running suture. The right lower circle (Tunneling) shows the right angle introduced into the tunnel once finished making sure that is not too tight.

The ureter to BMDL is performed in a running fashion manner with 6–0 PDS, first the lateral wall and then the medial wall. Halfway up the medial wall, the ureteral opening is checked with a right angle clamp, and the medial wall is finished. The two stay sutures are tied, not removed. The remaining detrusor muscle is closed over the anastomosis with interrupted sutures of 4–0 PDS, creating a muscular tunnel. Before placing the last interrupted 4–0 PDS, the tunnel is tested with the right angle clamp, making sure that the suture is not tight. The muscular tunnel is approximately 2.5cm in length and provides an anti-reflux effect (Fig 3).

Four main differences between our extravesical technique and those previously described by Lich-Gregoir [4, 5] and others [7–15] are outlined as follows: i) As described above, the bladder is mobilized as much as possible in order to allow it to move upward with the kidney (in case it moves upward) during abdominal closure. Its goal is to provide additional insurance against the risk of any rip or tear in the UVJ anastomosis from occurring, thereby decreasing urinary leak risk; ii) Our ureter has a posterior 2cm (more wide open) spatulation (thereby lowering ureteral stenosis risk), whereas three other extravesical approaches offer less spatulation, 0.5cm, 1cm and 1.5cm, respectively [10, 13, 14] During anastomosis of the non-spatulated portion of the ureter to the bladder mucosa, the total circumference of the ureter is included in the running suture, but during anastomosis of the spatulated ureter, only 50% of the ureteral orifice (original and spatulated orifice) or less is incorporated into the suture line. During inflammation and absorption of the absorbable suture, not all of the ureteral orifice is inflamed, thereby possibly further lessening the chance of developing ureteral stenosis [19, 20]; iii) The extravesical ureteral anastomosis is performed with running suture and incorporates both bladder mucosa and some (roughly 30% of) detrusor muscle. The 4 anchor stitches incorporate almost the complete thickness of the bladder wall. This gives added strength to the anastomosis in case of bladder fullness, urinary retention, or mobilization upward of the renal allograft during closure. In recipients having a thin detrusor muscle (i.e., mostly in patients who were transplanted due to polycystic kidney disease or received 8–10 years of dialysis pre-transplant), we were still able to incorporate the detrusor into the anastomosis to help strengthen it (again, further lowering the risk of a urinary leak from occurring); and iv) During suturing of the ureteral medial wall, a right angle clamp is introduced into the ureteral orifice to ensure that it is not stenosed or incorporated by the running suture, and the final stitch is tested with the right angle clamp, making sure that it is not too tight to cause stenosis. Use of a right angle clamp in this fashion was thought to further lower the risk of occurrence of an iatrogenic ureteral stenosis.

It was our belief that the use of this extravesical ureteroneocystostomy technique would not require the initial placement of a ureteral stent at the time of transplant. Additionally, the Foley catheter was planned to be removed on postoperative day 4–5 as long as there were no bladder issues.

### Data description

Between January 6, 2014 and September 9, 2019, 34 pediatric and 466 adult recipients of a kidney transplant performed consecutively by a single surgeon (G.C.) at the Miami Transplant Institute had this newly established “extravesical ureteroneocystostomy without ureteral stenting” technique performed. As Jackson Memorial Hospital is a teaching hospital, in all of these cases, the transplant fellow (or surgical resident) performed the other half of the anastomosis. The center institutional review board had approved this retrospective study of prospectively followed patients, and all patients (or their guardians) signed an informed consent prior to transplant. This study adheres to the ethical principles of the Helsinki Declaration (as revised in 2013).

All transplanted deceased donor kidneys had been connected to the LifePort Renal Preservation Machine® prior to transplantation using kidney perfusion solution (KPS-1®).

Patients who underwent other reimplantation techniques (performed by other transplant surgeons at our center) were not included, as their clinical outcomes were not available for this study. All patients in this study had a routine ultrasound of the kidney allograft performed during the first week post-transplant.

All patients received induction immunosuppression consisting of rabbit antithymocyte globulin (1mg/kg x 3 doses), basiliximab (20mg x 2 doses), and Solumedrol (500mg IV x 3 doses) during the first 4 days post-transplant. Maintenance immunosuppression consisted of tacrolimus (target 12-hour trough level: 4–8 ng/ml) and mycophenolic acid (720mg x 2 doses per day), along with corticosteroid avoidance [21].

Clinical outcomes that occurred during the first 12 months post-transplant were analyzed, including all urologic complications, delayed graft function (DGF, defined as the requirement for dialysis during the first week post-transplant), biopsy-proven acute rejection (BPAR, defined either as acute T-cell or antibody mediated rejection) [22], urinary tract infection (UTI) that required hospitalization, estimated glomerular filtration rate (eGFR) using the CKD-EPI formula [23], death-censored graft failure (defined as the return to permanent dialysis, graft nephrectomy, or re-transplantation, whichever occurred first), and death with a functioning graft (DWFG). Since the date of last follow-up was October 1, 2020, all patients were followed for at least 12 months post-transplant. Clinical outcomes occurring beyond 12 months post-transplant were not considered here.

All urologic complications that developed during the first 12 months post-transplant were recorded for this study. Suspected urologic complications were evaluated with renal ultrasonography, MAG-3 (mercaptoacetyltriglycine) scan, and/or antegrade nephrostogram. Urologic complications were treated by either percutaneous radiological procedures, surgery, or a combination of both. Suspected ureteral stenosis was evaluated along with serial monitoring for BK virus replication (in urine and blood).

Voiding cystourethrogram was not routinely performed, as routine evaluation for vesicoureteral reflux was not considered to be necessary in our transplant recipients; therefore, only symptomatic reflux was evaluated and considered as a urologic complication.

### Statistical analysis

The primary outcome variable for this study was the occurrence of a urologic complication (urinary leak or UVJ stenosis) during the first 12mo post-transplant. Secondary outcome variables included the (separate) occurrence of a urinary leak during the first 12mo post-transplant, (separate) occurrence of UVJ stenosis during the first 12mo post-transplant, other urologic complications that occurred during the first 12mo post-transplant such as hematoma, lymphocele, or graft thrombosis, DGF, a first BPAR that occurred during the first 12mo post-transplant, a UTI (or recurrent UTI) that required hospitalization during the first 12mo post-transplant, graft loss (death-censored and death-uncensored) during the first 12mo post-transplant, and DWFG during the first 12mo post-transplant.

Frequency distributions were determined for baseline (measured at the time of transplant) categorical variables, and the mean along with the standard deviation (SD) were calculated for baseline continuous variables. Tests of association between baseline categorical variables and the urinary complication outcomes were performed using Fisher’s (2-sided) exact test, as these were rare outcomes. Tests of association between baseline categorical variables and other outcomes, i.e., those having a greater frequency of occurrence such as first BPAR, UTI, and graft loss, were performed using Pearson (uncorrected) chi-squared tests. Mean values were compared using the ordinary t-test. In the attempt to avoid spurious associations, P-values < .01 were considered to be statistically significant.

Since there was no control group of patients available from our center in which the ureteroneocystostomy was performed using one of the more standard extravesical (Lich-Gregoir or modified Lich-Gregoir) techniques, with and without routine stent placement, instead, we performed a descriptive comparison with the results reported by other centers. Since tissue scarring and comprised blood flow can lead to any type of urologic complication (i.e., ureteral necrosis, ureteral leak, UVJ stenosis, and/or ureteral stricture), we thought the best (most reliable) outcome to use for comparison would be the reported overall percentage of patients/ureteroneocystostomies that developed any urologic complication (i.e., our primary outcome variable). Only extravesical ureteroneocystostomies performed by the other centers were considered, and center-specific stratification according to routine vs. no routine stenting (at the time of transplant) was also performed. In addition to including the overall meta-analysis results of Mangus and Haag [1], we included all studies (with complete data) published since that report which were included in the more recent meta-analysis of Alberts et al [6] or more recent review by Wilson et al [24]. A recently reported randomized trial [25] was also included as well.

## Results

### Patient demographics

Distributions of selected baseline variables are presented in Table 1. The percentage of deceased donor recipients was 64.6% (323/500), and a majority of recipients were either black (non-Hispanic), 35.6% (178/500), or Hispanic, 35.0% (175/500). Mean (+SD) recipient age at transplant was 50.7±17.5 (range: 2–82) years; mean donor age was 41.2±14.2 (range: 0.5–71) years. The percentage of donor kidneys having 1, 2, 3, and 4 donor arteries was 74.0% (370/500), 22.8% (114/500), 2.8% (14/500), and 0.4% (2/500), respectively. The percentage of patients that had pre-transplant bladder reconstruction was 1.2% (6/500).

**Table 1:** Distributions of selected baseline variables (N = 500).

Baseline Variable	Mean + SD if continuous
	Percentage with characteristic if categorical
Recipient Age (yr)	50.7 + 17.5 (N=500)
	[Median=54, Range: 2-82]
Recipient Age (yr):	
<1 8	6.8% (34/500)
18-49	31.2% (156/500)
>50	62.0% (310/500)
Recipient Gender:	
Female	370% (185/500)
Male	63.0% (315/500)
Recipient Race/Ethnicity	
Black (non-Hispanic)	35.6% (178/500)
Hispanic	35.0% (175/500)
White (non-Hispanic)/Asian^a^	29.4% (147/500)
Recipient BMI (kg/m^2^)	26.7 + 5.5 (N=500)
	[Median=26.2, Range: 14.1-41.4]
Recipient BMI (kg/m^2^):	
<25	40.4% (202/500)
25-29	33.0% (165/500)
>30	26.6% (133/500)
Underlying Cause of Renal Failure	
Diabetes Mellitus	27.0% (135/500)
Hypertension	24.2% (121/500)
PKD	8.2% (41/500)
Failed Renal Transplant	7.8% (39/500)
IgA Nephropathy	5.0% (25/500)
SLE	3.6% (18/500)
HIV	3.4% (17/500)
Glomerulonephritis	2.8% (14/500)
Hepatorenal Syndrome	2.4% (12/500)
Bilateral Renal Dysplasia	1.4% (7/500)
NSAID Nephropathy	1.2% (6/500)
Wegner’s Granulamatosis	1.0% (5/500)
Other	12.0% (60/500)
Recipient Pre-transplant Diabetes Mellitus	
No	69.6% (348/500)
Yes	30.4% (152/500)
Pre-transplant Bladder Reconstruction	
No	98.8% (494/500)
Yes	1.2% (6/500)
Preemptive Transplant	
No	79.8% (399/500)
Yes	20.2% (101/500)
Pre-transplant Time on Dialysis (mo)	41.7 + 41.8 (N=500)
	[Median=32.2, Range: 0-299.3]
Pre-transplant Time on Dialysis (mo):	
<12	31.6% (158/500)
12-59	38.4% (192/500)
>60	30.0% (150/500)
Donor Age (yr)	41.2 + 14.2 (N=500)
	[Median=41.5, Range: 0.5-71]
<18	3.8% (19/500)
18-49	62.4% (312/500)
>50	33.8% (169/500)
Number of Donor Arteries	
1	74.0% (370/500)
2	22.8% (114/500)
3	2.8% (14/500)
4	0.4% (2/500)
Donor Type	
Living	35.4% (177/500)
Deceased	64.6% (323/500)
DCD Status	
No	99.4% (497/500)
Yes	0.6% (3/500)
CIT (hr)	18.4 + 15.4 (N=500)
	[Median=20.8, Range: 0.2-69.5]
CIT (hr):	
<18	44.6% (223/500)
18-30	29.2% (146/500)
>30	26.2% (131/500)
Kidney Plus Other Organs Transplanted	
No	94.8% (474/500)
Yes	5.2% (26/500)
Re-transplant Status	
Primary	92.2% (461/500)
Re-transplant	7.8% (39/500)

Abbreviations: CIT, cold ischemia time; DCD, Donation after cardiac death; SE, Standard error.

^a^Asians comprised 6/147 of these patients.

### Urologic complications

Overall, the observed percentage of patients that developed a urologic complication was 1.4% (7/500). Of note, each of these 7 patients had received a deceased donor kidney. In total, the observed percentage of patients that developed an anastomotic urinary leak was 1.0% (5/500) (all 5 patients were adults); the times-to occurrence of these urinary leaks were 0.1, 0.5, 0.7, 1.7, and 2.9 months post-transplant, respectively. The 3 patients with the earliest times-to-urinary leak had documented ureteral necrosis. Each of these 3 patients were treated by open surgery with ureteral reimplantation and ureteral (double J) stent placement. The 4^th^ patient who developed a urinary leak at 1.7mo post-transplant subsequently developed a ureteral stricture one month later (at 2.7mo post-transplant). This patient was treated with placement of a temporary percutaneous nephroureteral stent with resolution of the urinary leak. The subsequent stricture was also successfully treated by a percutaneous approach including balloon dilatation of the stricture. The 5^th^ patient who developed a urinary leak at 2.9mo post-transplant was successfully treated by interventional radiology and nephroureteral stent placement. None of the 500 patients developed symptomatic reflux or had a renal ultrasound-showing moderate to severe hydronephrosis with wide-open ureter.

In total, the observed percentage of patients that developed UVJ stenosis was 0.6% (3/500) (2 of these 3 were pediatric cases). Times-to-occurrence of these ureteral stenoses were 0.6, 2.7, and 6.1 months post-transplant, respectively. One pediatric patient (age 16 years) with UVJ stenosis first occurring at 0.6mo post-transplant had urinary tract stones occluding the distal ureter and was initially misdiagnosed as a stricture. This patient was treated with ureteral reimplantation using the same ureteral technique without a ureteral stent. The 2^nd^ patient (an adult) had ureteral stenosis due to a stricture which developed at 2.7mo post-transplant, one month following the development of a urinary leak (described above). The other pediatric patient (age 9 years) with a ureteral stenosis/stricture occurring at 6.1mo post-transplant was initially treated by placement of a temporary percutaneous nephroureteral stent with balloon dilatation. However, after a failed interventional radiology attempt, this patient ultimately required ureteral reimplantation with ureteral stent placement.

In total, surgical repair was required for the 3 adults who developed ureteral necrosis (with a urinary leak) and the 2 pediatric cases who developed ureteral stenosis (urinary tract stones in 1 case; ureteral stricture in the other), yielding an overall incidence of 1.0% (5/500). The other 2 adult cases (as described above) were successfully treated by interventional radiology.

There was one notable association between having pre-transplant diabetes and risk of developing a urinary leak (Table 2): 0.0% (0/348) vs. 3.3% (5/152) of patients without vs. with a pre-transplant history of diabetes mellitus developed a urinary leak (P = .003). Four of the 5 diabetic patients who developed a urinary leak appeared to have a long pre-transplant history of diabetes (>20 years), including documented retinopathy, neuropathy, and cardiovascular disease in each of these 4 patients. No significant associations of any other baseline factors (such as recipient age, recipient race/ethnicity, recipient sex, donor age, and number of donor arteries) with the development of either a urinary leak or UVJ stenosis were observed (Table 2). For instance, the observed percentage of patients who developed a urinary leak during the first 12mo post-transplant was 1.1% (4/370) vs. 0.8% (1/130) for recipients of a donor kidney having only 1 vs. >2 donor arteries (P = 1.00). Similarly, the observed percentage of patients who developed UVJ stenosis during the first 12mo post-transplant was 0.8% (3/370) vs. 0.0% (0/130) for recipients of a donor kidney having only 1 vs. >2 donor arteries (P = .57).

**Table 2:** Univariable associations of selected baseline variables with the development of: i) a urinary leak (overall, 5/500), ii) UVJ stenosis (overall, 3/500), and iii) a urinary leak or UVJ stenosis (overall, 7/500) during the first 12 months post-transplant.

Baseline Variable	Observed Percentage Who Developed a Urinary Leak (during the first 12mo post-transplant)	P-value
Recipient Age (yr)		
<50	0.5% (1/190)	
>50	1.3% (4/310)	.65
Recipient Sex		
Female	1.6% (3/185)	
Male	0.6% (2/315)	.36
Recipient Race/Ethnicity		
Black (non-Hispanic)	1.1% (2/178)	
Hispanic	0.6% (1/175)	
White (non-Hispanic)	1.4% (2/147)	.76
Recipient Pretransplant DM		
No	0.0% (0/348)	
Yes	3.3% (5/152)	.003
Donor Age (yr)		
<50	0.6% (2/331)	
>50	1.8% (3/169)	.34
Number of Donor Arteries		
1	1.1% (4/370)	
>2	0.8% (1/130)	1.00
Baseline Characteristic	Observed Percentage Who Developed UVJ Stenosis (during the first 12mo post-transplant)	P-value
Recipient Age (yr)		
<50	1.6% (3/190)	
>50	0.0% (0/310)	.054
Recipient Sex		
Female	0.5% (1/185)	
Male	0.6% (2/315)	1.00
Recipient Race/Ethnicity		
Black (non-Hispanic)	1.1% (2/178)	
Hispanic	0.6% (1/175)	
White (non-Hispanic)	0.0% (0/147)	.43
Recipient Pretransplant DM		
No	0.6% (2/348)	
Yes	0.7% (1/152)	1.00
Donor Age (yr)		
<50	0.6% (2/331)	
>50	0.6% (1/169)	1.00
Number of Donor Arteries		
1	0.8% (3/370)	
>2	0.0% (0/130)	.57
Baseline Variable	Observed Percentage Who Developed a Urinary Leak or UVJ Stenosis (during the first 12mo post-transplant)	P-value
Recipient Age (yr)		
<50	1.6% (3/190)	
>50	1.3% (4/310)	1.00
Recipient Sex		
Female	1.6% (3/185)	
Male	1.3% (4/315)	.71
Recipient Race/Ethnicity		
Black (non-Hispanic)	1.7% (3/178)	
Hispanic	1.1% (2/175)	
White (non-Hispanic)	1.4% (2/147)	.91
Recipient Pretransplant DM		
No	0.6% (2/348)	
Yes	3.3% (5/152)	.03
Donor Age (yr)		
<50	1.2% (4/331)	
>50	1.8% (3/169)	.69
Number of Donor Arteries		
1	1.6% (6/370)	
>2	0.8% (1/130)	.68

The observed percentages of patients who developed a hematoma, lymphocele, and graft thrombosis during the first 12mo post-transplant were 1.8% (9/500), 0.4% (2/500), and 0.2% (1/500), respectively. Of note, all of the 9 hematomas occurred during the first month post-transplant. While the observed incidence of hematoma during the first 12mo post-transplant was higher among the 7 patients who developed (14.3%; 1/7) vs. did not develop (1.6%; 8/493) a urologic complication, this difference was not statistically significant (P = .12, Table 3). Of note, neither the 2 patients who developed a lymphocele nor the single patient who experienced graft thrombosis were among the 7 patients who developed a urologic complication.

**Table 3:** Univariable associations of the development of a urinary leak or UVJ stenosis (overall, 7/500) with the percentage developing another clinical outcome during the first 12 months post-transplant.

Percentage Developing Another Clinical Outcome	Developed a Urinary Leak or UVJ Stenosis		
	No (N=493)	Yes (N=7)	P-value
Hematoma	1.6% (8/493)	14.3% (1/7)	.12
DGF	13.4% (66/493)	28.6% (2/7)	.24
Acute Rejection	7.5% (37/493)	0.0% (0/7)	1.00
UTI	11.0% (54/493)	28.6% (2/7)	.18
Graft Failure (Death Censored)	1.8% (9/493)	14.3% (1/7)	.13
DWFG	2.6% (13/493)	14.3% (1/7)	.18

### Other clinical outcomes

The observed percentage of patients who developed DGF was 13.6% (68/500). While DGF incidence was observed to be higher among the 7 patients who developed (28.6%; 2/7) vs. did not develop (13.4%; 66/493) a urologic complication, this difference was not statistically significant (P = .24, Table 3).

The observed percentage of patients who developed an AR (either ACR or acute AMR) episode) during the first 12mo post-transplant was 7.4% (37/500), with 11/37 and 30/37 of these episodes occurring during the first month and 6mo, respectively. Of note, none (0.0%) of the 7 patients who developed a urologic complication had also developed an AR episode.

The observed percentage of patients who developed a UTI that required hospitalization during the first 12mo post-transplant was 11.2% (56/500), with 18/56 and 43/56 of these infections occurring during the first month and 6mo, respectively. In addition, the observed percentage who developed a recurrent UTI requiring hospitalization during the first 12mo post-transplant was 2.6% (13/500). While the observed incidence of UTI requiring hospitalization during the first 12mo post-transplant was higher among the 7 patients who developed (28.6%; 2/7) vs. did not develop (11.0%; 54/493) a urologic complication, this difference was not statistically significant (P = .18).

The overall percentage of patients who developed graft loss during the first 12mo post-transplant was 4.8% (24/500); 10 experienced death-censored graft failure, and 14 experienced death with a functioning graft. Causes of graft failure were as follows: primary nonfunction (N = 1), lower pole rupture (N = 1), hemorrhage from the arterial anastomosis (N = 1), graft thrombosis due to kidney allograft torsion (N = 1), acute rejection (N = 3), and infection/sepsis (N = 3). Among the 7 patients who developed a urologic complication, only 1 subsequently developed graft failure—this patient developed a urinary leak at 0.5mo post-transplant and then had graft nephrectomy at 5 days following this leak due to hemorrhage from the arterial anastomosis with negative cultures. While the observed incidence of death-censored graft failure during the first 12mo was higher among the 7 patients who developed a urologic complication, 14.3% (1/7) vs. 1.8% (9/493) among the other patients, this difference was not statistically significant (P = .13). In addition, while the observed incidence of death with a functioning graft during the first 12mo was higher among the 7 patients who developed a urologic complication, 14.3% (1/7) vs. 2.6% (13/493) among the other patients, this difference was also not statistically significant (P = .18).

Lastly, among 464 patients who were still alive with a functioning graft at 12 months post-transplant and had serum Cr measurements available at that time, the mean eGFR (+SD) was 79.2+32.4. No notable difference in mean eGFR at 12 months was observed between those who developed vs. did not develop a urologic complication, 81.3+36.1 (N = 5) vs. 79.2+32.4 (N = 459) (P = .88).

### Comparing our percentage who developed a urologic complication with other studies

Percentages of urologic complications that developed post-transplant, as reported by other kidney transplant studies of extravesical ureteroneocystostomies, stratified by routine stent placement (yes/no), appear in Table 4 [1, 25–38]. Our overall urologic complication incidence of 1.4% (7/500) is at least as good as every report (and better than most of the reports) shown in the table, including the 1.5% (6/407) incidence among Lich-Gregoir ureteroneocystostomies with routine stenting in 5 randomized trials combined as reported by Mangus and Haag [1]. In addition, in the recent retrospective review reported by Whang et al [36], in which a single transplant surgeon performed all of the Lich-Gregoir ureteroneocystostomies along with routine stenting, the overall incidence of urologic complications (excluding late complications) was 2.5% (95/3,856).

**Table 4:** Percentages of urologic complications that developed post-transplant, as reported by other kidney transplant studies of extravesical ureteroneocystostomies, stratified by routine stent placement (Yes/No).

Study Description Placed	Median/Mean	Percentage Developing a Urologic Complication	
	Follow-up (mo)	Routine Stent Placed	No Routine Stent
1. Mangus-Haag (2004) Meta-analysis [1]:			
5 Randomized Trials Combined	12^a^	1.5% (6/407)	9.0% (35/389)
44 Cohort Studies Combined	various^a^	3.2% (137/4,245)	4.8% (433/9,077)
2. Randomized Trial, Osman et al (2005) [26]:			
Lich-Gregoir	10.8	4.2% (2/48)	0.0% (0/50)
3. Randomized Trial, Ooms et al (2020) [25]:			
Lich-Gregoir	12	8.0% (8/100)	22.0% (22/100)
4. Cohort Study, Secin et al (2002) [27]:			
Lich-Gregoir	77	---------------	13.0% (54/416)
Shanfield “One-Stitch”	24	---------------	13.2% (21/159)
5. Randomized Trial, Zargar et al (2005) [28]:			
Lich-Gregoir	12	4.0% (4/100)	---------------
Taguchi “One-Stitch”	8	9.1% (4/44)	---------------
6. Cohort Study, Veale et al (2007) [29]:			
Lich-Gregoir	48	---------------	3.9% (14/360)
Shanfield “One-Stitch”	48	---------------	15.6% (55/353)
7. Cohort Study, Pacovsky et al (2007) [30]:			
Lich-Gregoir	30	---------------	16.0% (4/25)
Taguchi “One-Stitch”	27	---------------	18.2% (4/22)
8. Cohort Study, Lee et al (2007) [31]:			
Lich-Gregoir	33.4	---------------	5.9% (14/238)
Taguchi “One-Stitch”	37.8	---------------	15.1% (11/73)
9. Cohort Study, Georgiev et al (2007) [32]:			
Lich-Gregoir	12	5.9% (11/186)	17.9% (29/162)
10. Cohort Study, Tillou et al (2009) [33]:			
Lich-Gregoir	12	5.3% (22/412)	---------------
11. Cohort Study, Ameer et al (2011) [34]:			
Lich-Gregoir	34	---------------	6.1% (10/163)^b^
Taguchi “One-Stitch”	44	---------------	5.7% (12/209)^b^
12. Cohort Study, Kayler et al (2012) [35]:			
Lich-Gregoir	>6	3.9% (12/307)	4.6% (15/327)^c^
13. Cohort Study, Whang et al (2020) [36–38]:			
Lich-Gregoir	>24	2.5% (95/3,856)^d^	---------------

^a^The 12 months of follow-up shown for the 5 randomized trial results reported by Mangus and Haag^1^ was taken as the median follow-up among the 5 reported median follow-ups of the individual trials (as listed according to Mangus and Haag [1]): 3, 24, 24, 12, and 3 months post-transplant, respectively. Regarding the 44 cohort studies that were used in meta-analysis reported by Mangus and Haag [1], patient follow-up varied across the 44 different studies.

^b^Patients who developed a hematuria requiring intervention were not included as urologic complications here.

^c^A modified “full thickness” Lich-Gregior technique was used in the non-stented group.

^d^While UPJ obstruction and symptomatic vesicoureteral reflux were included as urologic complications in the Whang et al reports, yielding an overall incidence of 7.0% (269/3,856) for the occurrence of any urologic complication, these 2 types of complications were reported as occurring mostly late (beyond the first year) post-transplant. Thus, these 2 types of complications were excluded here.

## Discussion

More favorable outcomes following kidney transplantation have evolved over time, but the risk of developing urologic complications (especially in the early post-transplant period) has continued to be a major concern with its subsequent impact on patient morbidity and potentially even graft survival. Since 2014, one of the transplant surgeons at our center (G.C.) has used his own modified version of the Lich-Gregoir extravesical ureteroneocystostomy in 500 consecutively transplanted patients, without the placement of a ureteral stent at the time of transplant. The main goal of this anastomosis procedure (as described in the Materials and Methods section) was to try avoiding urologic complications altogether, because once a complication develops, it may require more than one (radiological and/or surgical) procedure to fully repair the problem. It was also believed that good results (in terms of minimizing the development of urologic complications) could be achieved without the need for selective or routine stent placement at the time of transplant. The observed percentage of patients in this rather large observational study who developed a urologic complication during the first 12mo post-transplant was exceptionally low, 1.4% (7/500), and surgical repair was only required in 1.0% (5/500). An additional advantage in performing stent-less ureteral anastomosis was that none of our patients required an extra surgical procedure simply for stent removal (except for those who required stent placement as treatment for the occurrence of a post-transplant urologic complication). With these results and the technical advantages offered by this extravesical ureteroneocystostomy technique, as described in the Materials and Methods section, we believe that further investigation of this technique is now warranted.

Mangus and Haag [1] in their meta-analysis demonstrated a difference in the post-transplant incidence of urologic complications seen in randomized, controlled studies of “routine” vs. “no routine” ureteral stent placement at the time of kidney transplant and extravesical ureteroneocystostomy: 1.5% (6/407) vs. 9.0% (35/389). The observed low complication rate of 1.5% among patients receiving “routine” ureteral stent placement may therefore serve as a high standard for comparison of results from other studies.

UTI is a common complication after kidney transplantation which accounts for approximately 45% to 72% of all infections occurring during the first 3mo post-transplant [39]. Abbott et al [40] reported a cumulative UTI incidence of 17% during the first 6mo post-transplant. Our observed incidence of UTI requiring hospitalization during the first 12mo post-transplant was 11.2% (56/500), acceptably low. With only 2.6% (13/500) of our cohort developing a recurrent UTI during the first 12mo post-transplant, these low UTI rates could be related (at least in part) to the absence of routine ureteral stent placement [1] and early Foley catheter removal [41].

Vesicoureteral reflux (VUR) is another complication of the ureteral anastomosis that can occur following kidney transplantation [42], and creation of an anti-reflux tunnel [3,7] at the time of the ureteral anastomosis can prevent its occurrence [43] In the technique described here, a 2.5cm tunnel was created with the inclusion of other detrusor layers, also making sure that it was not too tight to cause ureteral obstruction. As VUR is only investigated at our center if the patient becomes symptomatic, no further work-up was required in any of our patients who developed a recurrent UTI.

Since urinary leakage and particularly ureteral necrosis can be triggered by compromised ureteral blood flow, a technical aspect of the ureteral anastomosis is the preservation of periureteral tissue [44]. We observed 3 patients who developed ureteral necrosis, despite the fact their ureters had intact periureteral fat with periureteral vessels [45]. Some of the other transplanted ureters did not have intact periureteral fat with periureteral vessels, but ureteral necrosis was not observed in those cases. Thus, while the presence of intact ureteral wall blood flow is important in avoiding the occurrence of ureteral necrosis [46], it may still occur despite achieving (what appears to be) a good ureteral anastomosis with good periureteral tissue preservation.

Another factor related to ureteral blood flow and possibly an increased risk of developing a post-transplant urologic complication is the presence of multiple renal arteries in the donor kidney [44, 47]. While 26.0% (130/500) of our kidney transplant recipients received a donor kidney with more than one renal artery, its presence was not predictive of the risk of developing a post-transplant urologic complication (as shown in the Results).

Again, we want to emphasize the importance of trying to mobilize the bladder by giving it some mobility in case the kidney is pushed upward during abdominal wall closure. We also want to emphasize the technical aspect of including a layer of detrusor along with the bladder mucosa which adds strength to the anastomosis, with anchoring of the ureter distally and proximally to almost full thickness of the bladder. In the past, most of the urinary leaks observed at our center (when using the more conventional ureteral anastomosis) were at the heel of ureterovesical junction, i.e., at the connection of the ureter with the bladder mucosa (this area can be very thin and can therefore tear easily). Thus, it is our belief that by anchoring the ureter to almost full thickness eliminates one of the disadvantages of the more conventional extravesical ureteral anastomoses.

One study limitation is the fact that the results reported here were not based on a randomized controlled study, nor was there an available control group of transplanted patients at our center who received one of the more conventional extravesical ureteroneocystostomies. Certain baseline variables known to be associated with the risk of developing BPAR but not considered to be relevant for the risk of developing a urologic complication (i.e., number of HLA mismatches and cPRA at the time of transplant) were not recorded for this study. In addition, the favorable results reported here could be confounded with the fact that a single transplant surgeon (with many years of experience) performed each of the 500 consecutive transplant surgeries and ureteroneocystostomies contained in this report. On the other hand, the fact that all of the surgeries and ureteroneocystostomies were performed by one surgeon suggests that if the proposed technique is performed properly, then a minimization of the risk of developing a urologic complication is possible, and without the need for stent placement at the time of transplant.

In summary, we believe that the advantages in using this modified extravesical ureteroneocystostomy technique, i.e., mobilized bladder, longer spatulation of the ureter, inclusion of bladder mucosa with detrusor muscle layer in the ureteral anastomosis, and use of a right angle clamp in the ureteral orifice to ensure that it does not become stenosed, have clearly helped in lowering the early post-transplant risk of developing a urologic complication. Importantly, these excellent results were achieved without the need for ureteral stent placement at the time of transplant.

## Supporting information

S1 Data (XLSX)
